# Hypoxia adipose stem cell-derived exosomes promote high-quality healing of diabetic wound involves activation of PI3K/Akt pathways

**DOI:** 10.1186/s12951-021-00942-0

**Published:** 2021-07-07

**Authors:** Jie Wang, Hao Wu, Yixuan Peng, Yue Zhao, Youyou Qin, Yingbo Zhang, Zhibo Xiao

**Affiliations:** Department of Plastic and Aesthetic Surgery, The Second Affiliated Hospital of Harbin Medical University, Harbin, 150081 People’s Republic of China

**Keywords:** Diabetic wound, Hypoxia, Exosomes, ADSCs, PI3K/AKT

## Abstract

Refractory diabetic wounds can cause persistent inflammation and delayed healing due to hypoxia. Currently, no optimal solution is available. Exosomes of adipose stem cells (ADSCs-exo) may promote skin wound healing, however, molecular mechanisms remains mysterious. We found significantly enhanced survival and proliferation of adipose stem cells after hypoxia induction compared to normoxia. Here, we aimed to investigate if hypoxic adipose stem cells exosomes (HypADSCs-exo) participate in hypoxia adaptability and accelerate diabetic wound healing. Based on high-throughput sequencing, 215 microRNAs (miRNAs) were upregulated and 369 miRNAs downregulated in HypADSCs-exo compared to ADSCs-exo. Up-regulated miR-21-3p, miR-126-5p, miR-31-5p whereas down-regulated gene miR-99b and miR-146-a correlated with wound healing. According to Gene Ontology (GO) and Kyoto Encyclopedia of Genes and Genomes (KEGG), miRNAs might regulate cell metabolism, differentiation and Transforming growth factor-β (TGF-β) function. Consistently, HpyADSCs-exo could promote diabetic wounds healing and inhibit inflammation through PI3K/AKT signaling pathway. Collectively, HpyADSCs-exo can promote diabetic wound healing as an alternative strategy to improve wound healing.

**Supplementary Information:**

The online version contains supplementary material available at 10.1186/s12951-021-00942-0.

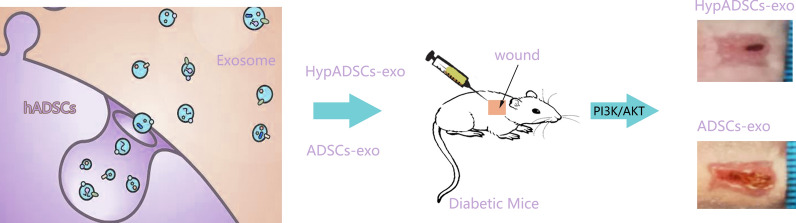

## Introduction

For diabetes, molecular mechanisms underlying wound healing are complex and involve dysfunction of multiple signaling pathways and processes. In particular, diabetic wound is susceptible to infection and scarring, while non-union or slow healing becomes a major culprit that seriously affects patients’ quality of life [1–4]. At initial stage of wound, cellular adaptability to hypoxia was decreased, resulting in persistent inflammation and substantially delayed healing [5]. Traditional treatment of diabetic trauma mainly focuses on late dressing, negative pressure, electrical stimulation, hyperbaric oxygen and skin grafting, but the treatment effect is not satisfactory [6]. At present, many new materials have been applied, bringing more possibilities for the complete cure of diabetic wounds, among which the application of exosome therapy shows a strong potential [7–11].

Adipose stem cells (ADSCs) hold a good prospect in wound repair [12]. Exosomes, extracellular vesicles with a diameter of 30–150 nm, mediate remote communication between cells. Exosomes can transferred to recipient cells, delivering proteins, RNAs and DNAs [13, 14]. Compared with cell therapy, exosomes may minimize immune mediated rejection and malignant transformation [15]. Hypoxia activates hypoxia inducible factor (HIF-1α), which regulates angiogenesis [11, 16, 17]. Interestingly, ADSCs-exo are involved in a wide range of biological processes by affecting tissue responses to injury, infection and diseases [18–23]. ADSCs secrete more exosomes under hypoxic environment, while HypADSCs-exo can improve blood perfusion and survival of transplanted tissues and reduce inflammatory filtration in adipose [24–27]. A variety of miRNAs in exosomes especially can promote diabetic wound healing [28, 29]. Although evidence on high-quality skin wound healing is still lacking at this stage, these results provide a new perspective for HypADSCs-exo in soft tissue repair.

Our study show that ADSCs-exo and HypADSCs-exo to identify differentially expressed miRNAs with performed high-throughput sequencing. Notably, HypADSCs-exo induced proliferation, collagen metabolism and migration through PI3K/AKT signaling pathway in human skin fibroblasts. Furthermore, we established a diabetic wound model in nude mice to analyze speed and quality of wound tissue healing, and to explore molecular mechanism underlying beneficial effects of HypADSCs-exo. Our study provides a promising strategy for cell-free therapy.

## Materials and methods

### Cell culture

Subcutaneous adipose and skin tissue samples were harvested from the abdomen of women (aged 20–50 years) who received liposuction from January to May 2020 at the Second Affiliated Hospital of Harbin Medical University. Informed consent was obtained from each subject. Acquisition of human subcutaneous fat aimed to establish primary culture of adipose stem cells. The cell pellet was resuspended in Dulbecco’s Modified Eagle Medium F12 (DMEM/F12; Corning, New York, USA) supplemented with 10% fetal bovine serum (FBS; Gibco, Thermo Fisher Scientific, Rockville, MD, USA) and 100 IU penicillin/100 mg/mL streptomycin (Solarbio, Beijing, China), and cultured in a humidified 5% CO_2_ atmosphere at 37 °C. Similarly, we conducted primary cultures of human fibroblasts (HF).

### Isolation and analysis of exosomes

Human ADSCs (hADSCs) at 70–80% confluence were washed with PBS and cultured in microvascular endothelial cell growth medium‐2 media deprived of FBS with supplement of 1× serum replacement solution (PeproTech) for 24 h. Similarly, the HADSCs were subjected to hypoxia conditions (1% O_2_/5% CO_2_/94% N_2_) for 24 h to obtain the cell supernatant and isolate the HypADSCs-exo. Then, the cell supernatant was then collected and centrifuged at 300*g* for 10 min, followed by centrifugation at 2000×*g* for 10 min at 4 °C. Then, the cell supernatant was filtered with a 0.22 μm sterile filter (sterile micropores, Burlington). We follow the instructions to add the ExoQuick‐TC reagent. After centrifugation at 1500*g* for 30 min, discarding the cell supernatant and resuspend the exosome‐containing pellet in PBS. Exosomes were either applied immediately for experiments or stored at − 80 °C. Dimensions of purified exosomes were determined with NanoSight LM10 (Malvern Instruments) nanoparticle tracking system. Levels of HSP70 and CD9 proteins were detected by Western blotting (abcam, USA). ADSCs-exos were labeled using PKH26 Red Fluorescent Cell Linker Kits according to the manufacturer’s instructions (Sigma, USA). Specific concentrations of proteins in exosomes were assessed with bicinchoninic acid assay kits (Beyotime, China). Ultrastructure of extracellular vesicles was analyzed by Transmission electron microscopy (TEM) with Libra 120 instrument (Zeiss).

### MiRNAs high-throughput sequence

Exosomas miRNAs was isolated using a commercially available total exosomes RNA isolation kit (Qiagen’s exoRNeasy Serum Plasma Kit). RNA integrity was verifified on a Bioanalyzer 2100 using an Agilent RNA 6000 Nano Assay. QPCR’s KAPA Biological System Library Quantization Kit for quantitative sequencing applications (Kapa Biosystems, Inc., Woburn, Mass). Each library was diluted to a final concentration of 10 nM and pooled equimolar before clustering. Also, to meet the sample compliance requirements, we performed paired end sequencing and analysis of gene coverage and thermal graph. Quality analysis of gene coverage graph and thermal graph. GO and KEGG analyses for differentially expressed miRNAs identified downstream genes regulated by miRNAs (*P* < 0.05, |log2 (fold change)|> 1).

$$ P = 1 - \sum\limits_{{i - 0}}^{{m - 1}} {\frac{{\left( {\begin{array}{*{20}c}    M  \\    i  \\   \end{array} } \right)\left( {\begin{array}{*{20}c}    {N - M}  \\    {n - i}  \\   \end{array} } \right)}}{{\left( {\begin{array}{*{20}c}    N  \\    n  \\   \end{array} } \right)}}} $$

### RNA extraction and real-time PCR

Total RNA was isolated from hADSCs after treatments using RNAiso Plus (TaKaRa Biotechnology, Shiga, Japan) according to the manufacturer’s instructions. Reverse the separation RNA to cDNA. Next, mRNAs expression was measured via real-time PCR in an ABI Prism 7500 Sequence Detection System (ABI, CA, USA) using SYBR Premix ExTaq (TaKaRa Biotechnology) (Additional file 2: Table S1).

### Human fibroblasts (HF) treatment

HF(5 × 10^6^ cell) treated with ADSCs-exo, HypADSCs-exo (100 μg/mL) or PBS (control). Next, we treat the cells with the PI3K/AKT inhibitor LY294002 (50 nM, boss from MedChem Express, Monmouth Junction, NJ, USA). Cells were harvested 30 min after treatment for western blotting and after 48 h or for Edu assays.

### Cell proliferation assay

Cell proliferation was detected using the Edu colorimetric immunoassay kit (Cell Proliferation ELISA, Roche Diagnostics, Germany). Cell proliferation was expressed as the mean percentage of the control values (set at 100%).

### Western blot analysis

Total separated protein components, control, ADSCs-exo, HypADSCs-exo (100 μg/mL) or PBS induced HF 48 h and cell lysis buffer treated 15 min. The lysate centrifuged at 12,000 rpm at 4 °C for 15 min. Then, 20 μg was separated by 15% SDS/PAGE and transferred onto polyvinylidene diflfluoride membranes (Millipore, Mississauga, Canada). The membranes were blocked in milk for 1 h and were incubated overnight at 4 °C with primary antibodies GAPDH (ab181602, Abcam), Type I collagen (COL I) (ab34710, Abcam), Type III collagen (COL III) (ab184993, Abcam), P-AKT (13038, Cell Signaling Technology), AKT (9272, Cell Signaling Technology) in blocking solution at a compatible dilution. Membranes were treatment with anti-rabbit/anti-mouse secondary antibodies (Abcam) in blocking solution at a dilution of 1:8000 and incubated for 1 h at RT. The bands were detected using an ECL kit (Beyotime BioTech, Shanghai, China) according to the manufacturer’s protocol. The film signals were digitally scanned with ImageQuant LAS 4000 mini machine (GE).

### Cell migration

6-well plate plated into 2 × 10^5^ human fibroblasts (HF) and scrape smooth edges with the 200 μl fluid head. The serum-free F-12 medium (500 μl) with exosomes (50 μg/ml ADSCs-exo, HypADSCs-exo) or PBS was added to the well. Shooting with a Lecka inverted microscope after 0, 12, 24 h, Migration area is calculated after 0, 12, 24 h (Leica Microscopy for camera).

Similarly, the the bottom of the transwell plates add to exosomes. HF were plated in serum-free medium in the upper chamber of a Transwell. After 24 h, remove upper membrane cells with cotton swab and rinse. We used 0.1% crystal violet staining and Lecka inverted microscope for photos.

### Diabetic wound healing evaluation

BALB/c nude mice (4 weeks old) were obtained from the Second Affiliated Hospital of Harbin Medical University and approved by IACUC and fed with 45% high fat diet for 5 weeks. After fasting for 12 h without food and water, the mice were intraperitoneally injected with streptozotocin (35 mg/kg in 0.1 M citrate-buffered saline, pH 4.5) to induce Diabetes Mellitus (DM). Glucose was measured with blood sugar test paper (Roche, Basel, Switzerland). Glucose level > 16.7 mmol/L indicated DM. To establish a stable animal model of DM, diabetic nude mice were re-fed with high-fat diet for 4 week and blood glucose levels were reconfirmed before wound formation. Anesthesia was performed with intraperitoneal injection of 10% chloral hydrate solution (250 μl/100 g). A square full thick skin injury (0.8 cm × 0.8 cm) was produced on the back of each nude mice. Then diabetic nude mice were randomly divided into three groups, respectively, in which ADSCs-exo (DW + exo), HypADSCs-exo (DW + hexo) (2 mg in 100 μL PBS) or 100 μL PBS (DW) were subcutaneously injected into four mid-points of the wound edge. Normal nude mice (Ctrl) with normal diet and water received the same wound operation. The wound was photographed on days 0, 3, 7 and 14, respectively after surgery to observe the healing process. Image J software was used to analyze wound size on days 0, 3, 7 and 14, respectively.

### Histology

Tissue specimens were fixed with 4% paraformaldehyde solution and then embedded in paraffin. Sections were sectioned and cultured overnight with primary antibodies against β-actin, CD31, COLIII, IL-6, or TGF-β overnight at 4 °C and with secondary antibodies (Abcam) for 1 h at 37 °C. The sections were stained with 3,3-diaminobenzidine and counter stained with hematoxylin. Immunofluorescence staining on COLI (1:100; ab34710) was performed. Hematoxylin–eosin (H&E) and Masson's trichrome staining were conducted. The expression levels of COLI, TGF-β, PDGF and VEGF mRNA in tissues of nude mice were detected by qRT-PCR. Get images with a Lecka microscope and randomly select five different fields.

### Statistical analysis

All data were used of GraphPad Prism13.0 software and Image J analyses and expressed as mean ± standard deviation (SD). One-way ANOVA and *t*-test was used for comparison of two or more groups. *P* < 0.05 was considered to be statistically significant.

## Results

### Characterization of ADSCs-exo

**Fig. 1:**
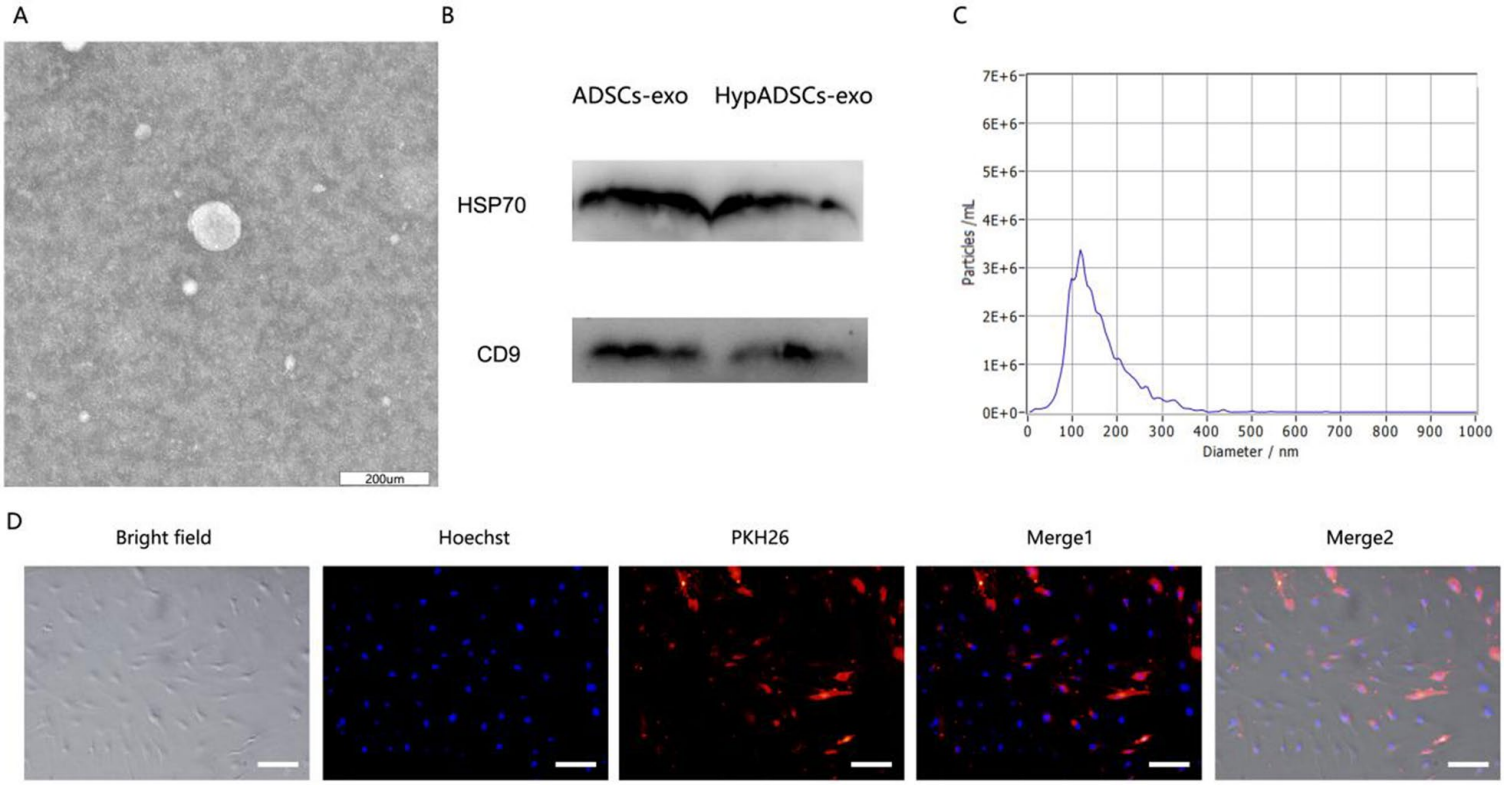
Characterization of ADSCs-exo. **A** Morphology observed under transmission electron microscope. **B** Particle size distribution. **C** Western blot was used to detect exosomes surface markers. **D** Fluorescent microscopy analysis of PKH26-labeled ADSCs-exo internalization by tenocytes. Bars, 100 μm

Under transmission electron microscopy, ADSCs-exo were round vesiculas (Fig. 1A), expressing specific markers HSP70 and CD9 (Fig. 1B), with size of 110 nm (Fig. 1C). We observed red PKH26-labeled exosomes around the nucleus, indicating that ADSCs-exo were internalized by fibroblasts (Fig. 1D).

### Expression profiles of miRNAs in ADSCs-exo and HypADSCs-exo

**Fig. 2:**
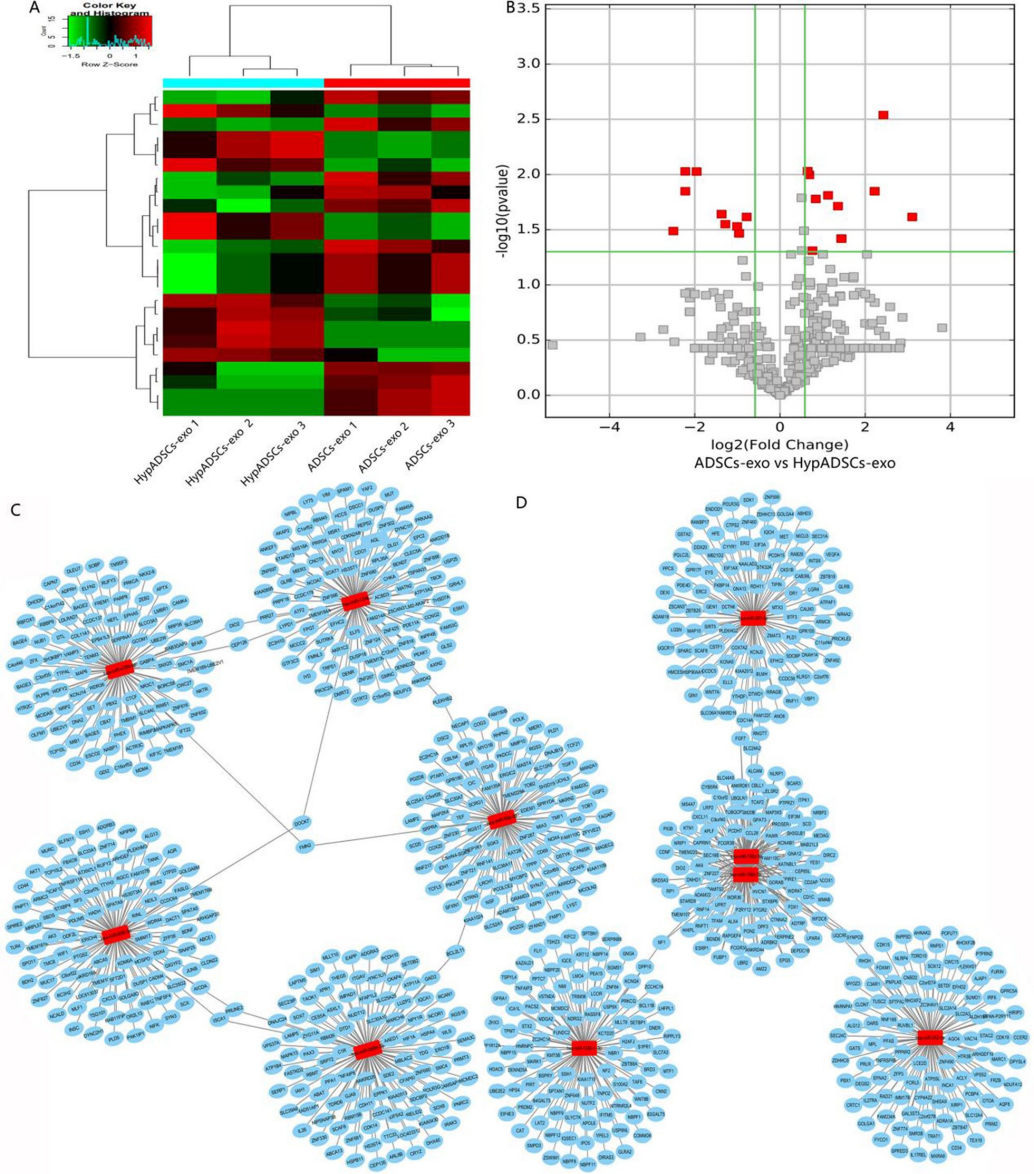
High-throughput sequencing analysis ADSCs-exo and HypADSCs-exo miRNA differential expression. **A** Clustered heat map of differentially expressed miRNAs depicting up and down regulated miRNAs. **B** Volcano plot of differentially expressed miRNAs depicting up and down regulated miRNAs. **C**, **D** Schematic representation of the predicted target genes and corresponding cellular functions of the miRNAs (**C** up-regulated miRNAs and **D** down-regulated miRNA) senriched in exosomes

**Fig. 3:**
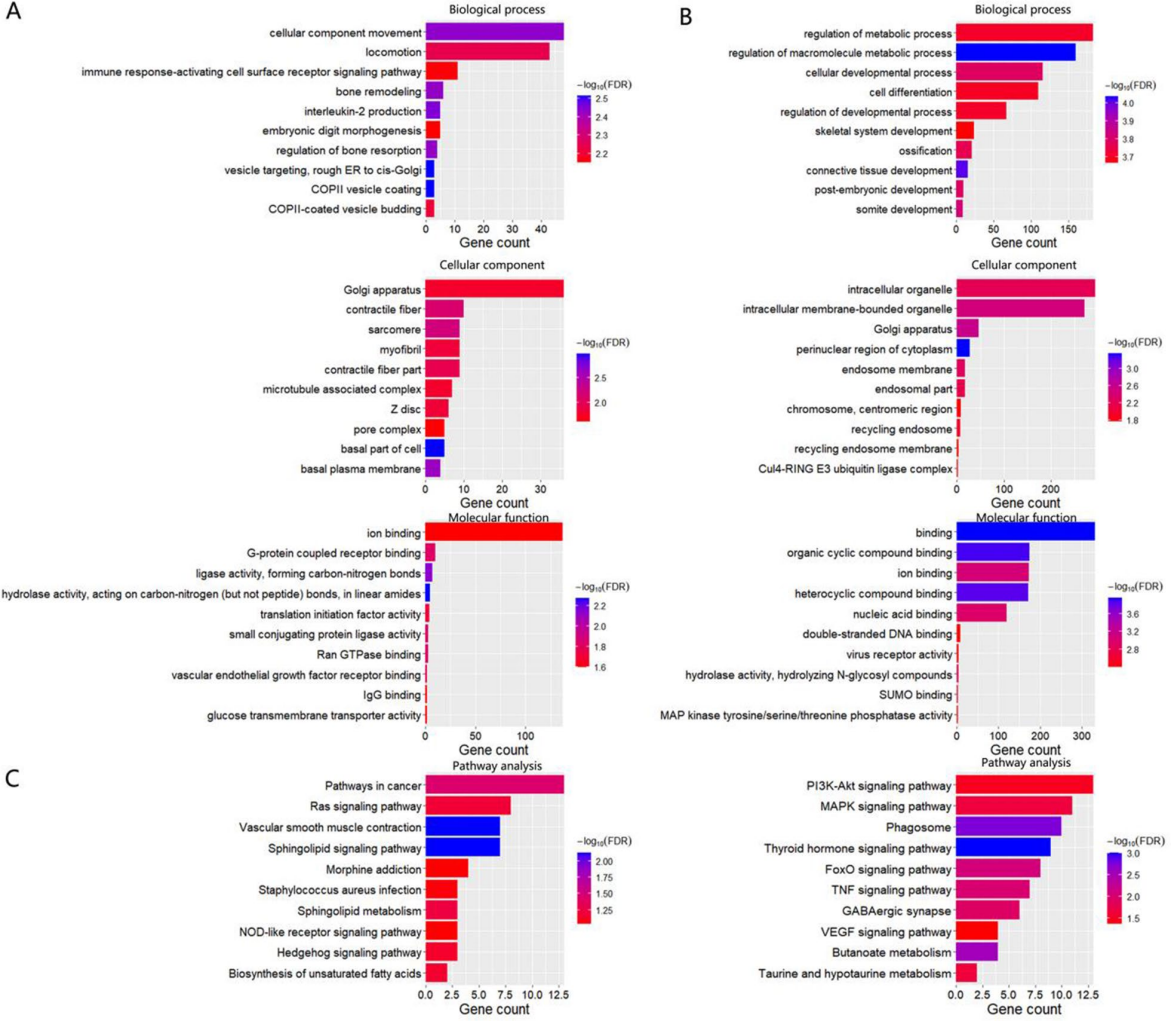
GO and KEGG enrichment of differentially expressed miRNAs. **A** GO enrichment of down-regulation expressed miRNAs. **B** GO enrichment of up-regulation expressed miRNAs. **C** Significant terms in KEGG pathways, the left is down-regulation, the right is up-regulation. n = 3

Differential expression profiles of miRNAs were described in Fig. 2. As shown in Fig. 2A, B, clustered heat map and volcano plot of differentially expressed miRNAs depicted up- and down-regulated miRNAs in HypADSCs-exo, compared to ADSCs-exo. 369 miRNAs were downregulated whereas 215 upregulated as presented by Volcano Plot filtering. Downstream genes of differentially expressed miRNAs were analyzed with GO and KEGG. GO enrichment was shown in Fig. 3A, B. The most common biological process was regulation of macromolecule metabolism (GO:0060255). The most enriched cellular component was perinuclear region of cytoplasm (GO:0048471). The most enriched molecular function was binding (GO:0005488). As shown in Fig. 3C, KEGG pathway analysis revealed thyroid hormone signaling pathway as the most enrichment factor.

### HypADSCs-exo promotes fibroblast proliferation and migration in vitro

**Fig. 4:**
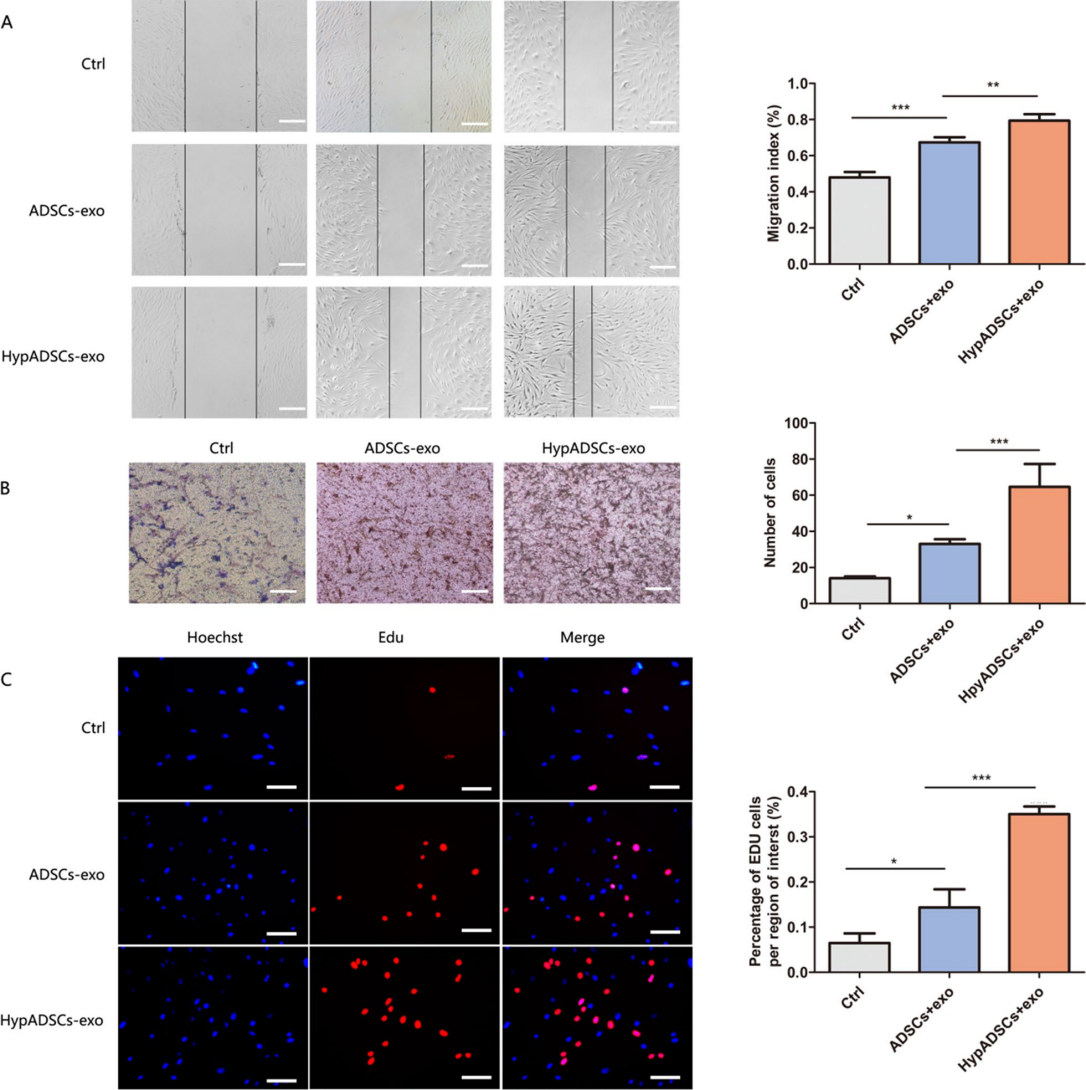
HypADSCs-exo significantly promote the proliferation and migration of HF. **A**, **B** The migration ability of HF treated with HypADSCs-exo, measured by scratch and Transwell test assays. **C** The proliferation of cells by Edu assays. Data are represented as mean ± SD. n = 3. *P < 0.01, **P < 0.001, ***P < 0.0001. Scale bars, 100 μm.

Based on scratch assay, we found that the migration of HypADSCs-exo component fibroblasts increased significantly (Fig. 4A). Similar result was demonstrated by transwell assay (Fig. 4B). HypADSCs-exo significantly promoted fibroblast proliferation (Fig. 4C).

### HypADSCs-exo regulate fibroblast chemokines and extracellular matrix formation

**Fig. 5:**
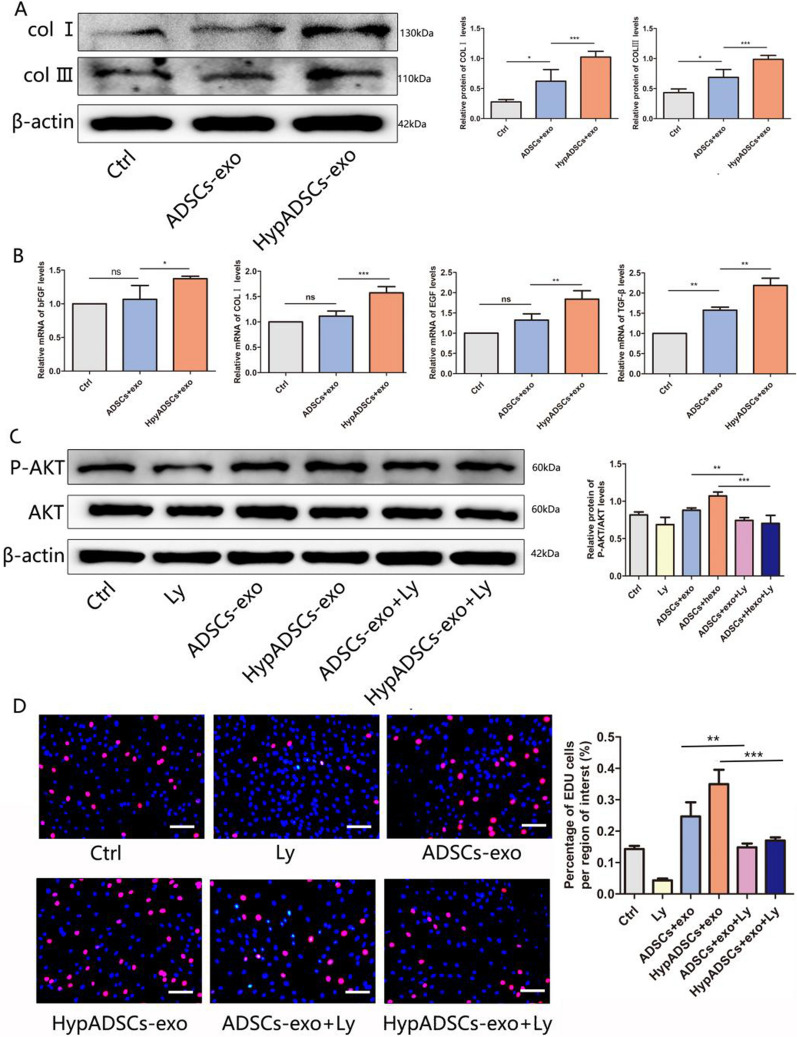
HypADSCs-exo promote the expression and secretion of extracellular matrix and growth factors in HF. **A** Analysis of protein expression levels in treated HF. **B** QRT-PCR analysis of mRNA levels, including bFGF, PDGF, COLI, EGF. Data are represented as mean ± SD. *, vs ADSCs-exo; #, vs Ctrl. **C** Western-blot analysis of protein levels of P-AKT induced by different concentrations of control, ADSCs-exo and HypADSCs-exo. Ly294002 inhibit the activation of PI3K/ AKT induced by control, ADSCs-exo and HypADSCs-exo, respectively. **D** Edu assay showed that HypADSCs-exo-mediated HF proliferation was suppressed by inhibitors Ly294002, compared with ADSCs-exo. Data are represented as mean ± SD. *P < 0.01, **P < 0.001, ***P < 0.0001. Scale bars, 100 μm

Expression of HypADSCs-exo proteins in fibroblasts was significantly increased (Fig. 5A). ADSCs-exo gene expression of COLI, TGF-β, EGF and bFGF was significantly increased in fibroblasts (Fig. 5B). As a result, HypADSCs-exo regulated the production of extracellular proteins and chemokines in fibroblasts and exerted potential effects on angiogenesis.

### Regulation of PI3K/AKT signaling in fibroblast

PI3K/AKT signaling pathway promotes cell proliferation, migration and wound healing. To investigate if PI3K/AKT could regulate behaviors of fibroblasts, we pretreated fibroblasts with Ly294002 (PI3K/AKT inhibitor). Accordingly, AKT phosphorylation induced by HypADSCs-exo was significantly inhibited (Fig. 5C). Upon inhibiting PI3K/AKT signaling, HypADSCs-exo mediated proliferation of fibroblast was significantly attenuated (Fig. 5D). Thus, proliferation and migration of fibroblasts regulated by HypADSCs-exo might be dependent on PI3K/AKT signaling.

### HypADSCs-exo accelerate skin wound healing in diabetic mice

**Fig. 6:**
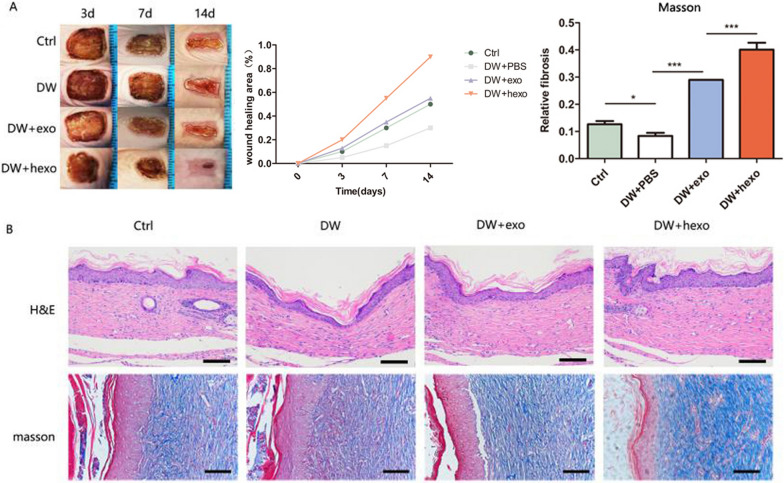
Observation of wound healing quality and velocity after operation. **A** After HypADSCs-exo treatment 0, 3, 7 and 14 days were taken. **B** HE staining of tissue wounds 14 days after operation. Masson staining of tissue wounds 14 days after operation. Data are represented as mean ± SD. *P < 0.01, **P < 0.001, ***P < 0.0001. Scale bars, 100 μm

We established a diabetic wound model with nude mice. From the images taken, the wound healing of DW-hexo group was significantly better than that of DW-hexo, DW or Ctrl (Fig. 6A). In DW-hexo group, wounds were almost completely closed on day 14. As for wound closure rate, HypADSCs-exo treated wounds contracted much faster than ADSCs-exo treated wounds on days 7 and 14. Additionally, HypADSCs-exo treated skin wounds had complete re-epithelialization and cuticle covering on the epidermis as demonstrated by blue skin fibers (Fig. 6B). By contrast, skin wounds treated with ADSCs-exo exhibited less re-epithelialization.

### HypADSCs-exo regulate inflammatory factors, chemokines and extracellular matrix formation in diabetic mice

**Fig. 7:**
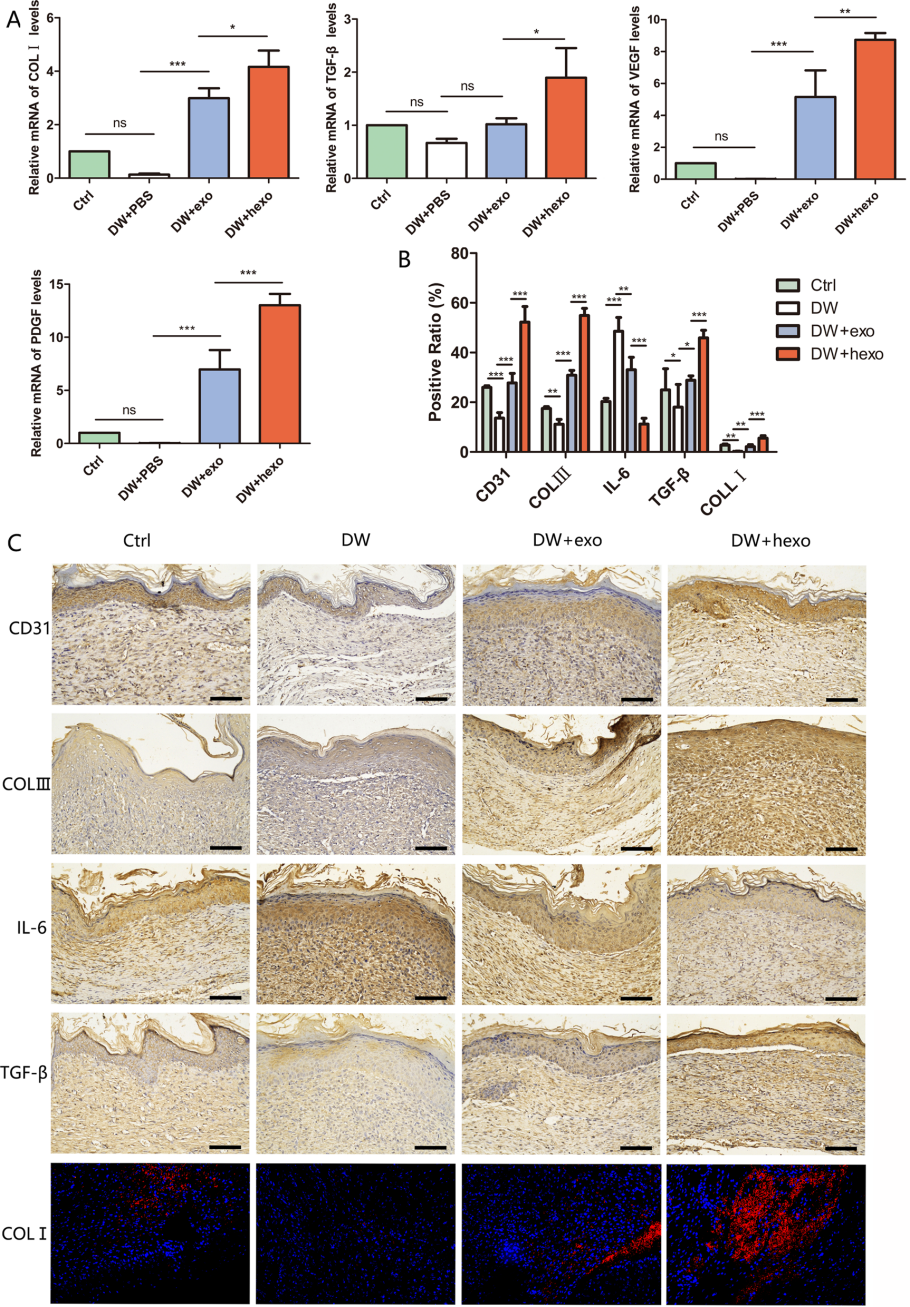
Genetic and histological analysis of wound healing in diabetes **A** QRT-PCR analysis of wound tissue mRNA level 14 days after operation, including COLI, TGF-β, VEGF, PDGF. **B**, **C** Expression of CD31, COLIII, IL-6, TGF-β, COLI in wound tissue was observed 21 days after operation. Data are represented as mean ± SD. *P < 0.01, **P < 0.001, ***P < 0.0001. Scale bars, 100 μm

In wound tissue of DW-hexo group, upregulated expression of collagens and growth factors (TGF-β, PDGF, COLI and VEGF) in skin tissue cells could accelerated wound healing (Fig. 7A). Potential effects of DW-hexo on stromal-related factors CD31, TGF-β, COLI, COLIII as well as inflammatory factor IL-6 in diabetic wound were investigated. Interestingly, expression of CD31, TGF-β, COLI and COLIII was upregulated while IL-6 was downregulated in DW-hexo treatment group at week 2 (Fig. 7B), in comparison with DW-exo, DW or Ctrl (Additional file 1: Figure S1).

## Discussion

Incidence and prevalence of diabetes keep rising globally [30]. Because of slow healing and even non-healing, diabetic wound has brought a great challenge to therapy [31]. Diabetic patients with foot ulcers are 2.5 times more likely to die in 5 years [32]. Therefore, it is necessary to explore molecular mechanisms underlying diabetic wound healing and to identify effective treatment of diabetic wound. Importantly, ADSCs together with conditioned medium may promote skin wound healing and regeneration [33, 34]. Compared with adipose stem cell transplantation, ADSCs-exo has greater potential, and ADSCs-exo under hypoxia has advantages in promoting angiogenesis and bone healing compared with that under normorxia [35, 36]. In this study, we have demonstrated that HypADSCs-exo promotes fibroblast proliferation and migration by activating PI3K/AKT pathway, which can accelerate healing of diabetic wounds.

In the early stage of diabetic healing, cells adapt to hypoxia; while in the late stage, inflammatory response and growth factors directly affect the process of wound healing [37–39]. Therefore, we analyzed differential expression of miRNAs and related pathways, cell functions and target proteins between hypoxic exosomes and normoxic exosomes with high-throughput sequencing. 215 miRNAs were upregulated whereas 369 downregulated in hypoxic exosomes compared with normoxic exosomes. Upregulated miR-21-3p/miR-126-5p/miR-31-5p whereas down-regulated miR-99b/miR-146-a might play a role in promoting fibroblast proliferation and migration, as well as in regulating immune response by activating targeted signaling pathways [40, 41]. GO and KEGG indicate regulatory effects of adipose stem cell exosomes on cell metabolism, differentiation and TGF-β secretion under hypoxia, partially through activating PI3K/AKT and MARK pathways. Thus, we propose HypADSCs-exo can accelerate diabetic wound healing. Consistently, HypADSCs-exo can regulate inflammation and extracellular matrix secretion, partially through PI3K/AKT signal pathway, to accelerate wound healing in diabetes. This finding provides a new solution for refractory diabetic wounds.

Fibroblasts are the important effector cells in skin wounds [3]. Skin fibroblasts interact with keratinocytes, adipocytes, mast cells and extracellular matrix (including collagen) [42, 43]. Accordingly, HypADSCs-exo induced proliferation and migration of fibroblasts. HypADSCs-exo also increases production of extracellular matrix and growth factors. PI3K/AKT signaling pathway is involved in regulating cell proliferation and migration, while exosomes can activate PI3K/AKT for survival [44, 45]. Notably, HypADSCs-exo induce AKT fast channels, which can be weakened by Ly294002, suggesting that survival-promoting signals in fibroblasts can weaken HypADSCs-exo fast channels to improve cell viability. ADSCs-hexo induced fibroblast proliferation and migration partially depends on PI3K/AKT pathway. Besides, TGF-β stimulates fibroblast proliferation in coordination with bFGF [46]. EGF as a chemokine promotes fibroblast proliferation and migration [47]. In our study, TGF-β, EGF, COLI, bFGF induced by HypADSCs-exo were increased significantly in fibroblasts. HypADSCs-exo may regulate the expression of multiple growth factors, to promote the proliferation and migration of fibroblasts, as well as angiogenesis.

A diabetic wound model using nude mouse was developed and treated with HypADSCs-exo in vivo. It’s noteworthy that HypADSCs-exo can accelerate high-quality healing of diabetic wounds compared with ADSCs-exo. It’s well-known that extracellular matrix plays a supporting and elastic role in wound healing and regeneration, depending on the secretion of collagen I and III. HypADSCs-exo may regulate extracellular matrix formation, thereby accelerating wound healing in diabetes. The expression of IL-6 was decreased in wound tissue of HypADSCs-exo treated diabetic nude mice, while the expression of VEGF was increased. Our study suggests that HypADSCs-exo may modulate the remodeling of external matrix in diabetic wound healing and thus improve scar fibrosis. These results suggest that HypADSCs-exo may play a key role in extracellular matrix remodeling during wound healing and thus partially improving scar fibrosis. We detected a significant increase in TGF-β, COLI, PDGF and VEGF expression in HypADSCs-exo treated diabetic mice. In this regard, VEGF has the potential to initiate angiogenesis and promote wound healing [48]. PDGF released by platelets binds to fibroblast surface receptors during wound repair [49]. HypADSCs-exo may promote skin regeneration by regulating the secretion and expression of growth factors. Meanwhile, the expression of CD31 was up-regulated, indicating increased angiogenesis. Taken together, HypADSCs-exo may accelerate high-quality healing of diabetic wound, which provides the possibility for clinical application of HypADSCs-exo.

Nevertheless, our study has some limitations. For example, potential effects of specific miRNAs derived from HypADSCs-exo on fibroblasts need further investigation. Moreover, PI3K/AKT signaling may not be the only pathway in HypADSCs-exo that affects fibroblasts, which requires illustration. Furthermore, clinical application of HypADSCs-exo requires more accurate injection dose and time to achieve an optimal therapeutic efficacy.

## Conclusion

Collectively, HypADSCs-exo may accelerate the rate of diabetic wound healing, improve the quality of wound healing whereas inhibit inflammation. HypADSCs-exo promotes the proliferation and migration of fibroblasts by activating the PI3K/Akt pathway, and thus enhancing the secretion of vascular growth factors and extracellular matrix. These results provide a new treatment strategy by applying HypADSCs-exo in diabetic wound repair.

## Supplementary Information

**Additional file 1: Figure S1.** (A) ROS generation was evaluated by fluorescence microscopy, and a stronger red fluorescence intensity indicates a higher production of ROS. (B) HIF-1α protein levels increased after HypADSCs-exo treatment of HF. Data are represented as mean ± SD. n = 3. *P < 0.05, **P < 0.01. Scale bars 100 μm.

**Additional file 2: Table S1. **Primer sequences of the study.

## Data Availability

The data that support the findings of this study are available from the corresponding author upon reasonable request.

## Abbreviations

ADSCs-exo: Adipose stem cells exosomes
HypADSCs-exo: Hypoxic adipose stem cells exosomes
bFGF: Basic fibroblast growth factor
COLI: Collegen I
COLIII: Collegen III
Ctrl: Control
DW: Diabetic wound
DW + exo: Diabetic wound exosomes
DW + hexo: Diabetic wound hypoxic exosomes
DMEM/F12: Dulbecco’s Modified Eagle Medium F12
FBS: Fetal bovine serum
GO: Gene ontology
HIF-1α: Hypoxia inducible factor-1α
HF: Human fibroblast
H&E: Hematoxylin and eosin
IRB: Institutional review board
IL-6: Interleukin-6
KEGG: Kyoto encyclopedia of genes and genomes
SDF-1: Stromal cell-derived factor-1
TGF-β: Transforming growth factor-β
TEM: Transmission electron microscopy
P-AKT: Phosphate-AKT
PMSF: Phenylmethanesulfonyl fluoride
PDGF: Platelet derived growth factor
qRT-PCR: Quantitative reverse transcription-polymerase chain reaction
SD: Standard deviation
VEGF: Vascular endothelial growth factor
