# Supplementary material for: Hypoxia adipose stem cell-derived exosomes promote high-quality healing of diabetic wound involves activation of PI3K/Akt pathways
Source: J Nanobiotechnology. 2021 Jul 7;19:202. doi: 10.1186/s12951-021-00942-0 (PMC8261989; doi:10.1186/s12951-021-00942-0)
Supplement: Supplementary file 1 — Additional file 1: Figure S1. (A) ROS generation was evaluated by fluorescence microscopy, and a stronger red fluorescence intensity indicates a higher production of ROS. (B) HIF-1α protein levels increased after HypADSCs-exo treatment of HF. Data are represented as mean ± SD. n = 3. *P < 0.05, **P < 0.01. Scale bars 100 μm. [file 12951_2021_942_MOESM1_ESM.docx]

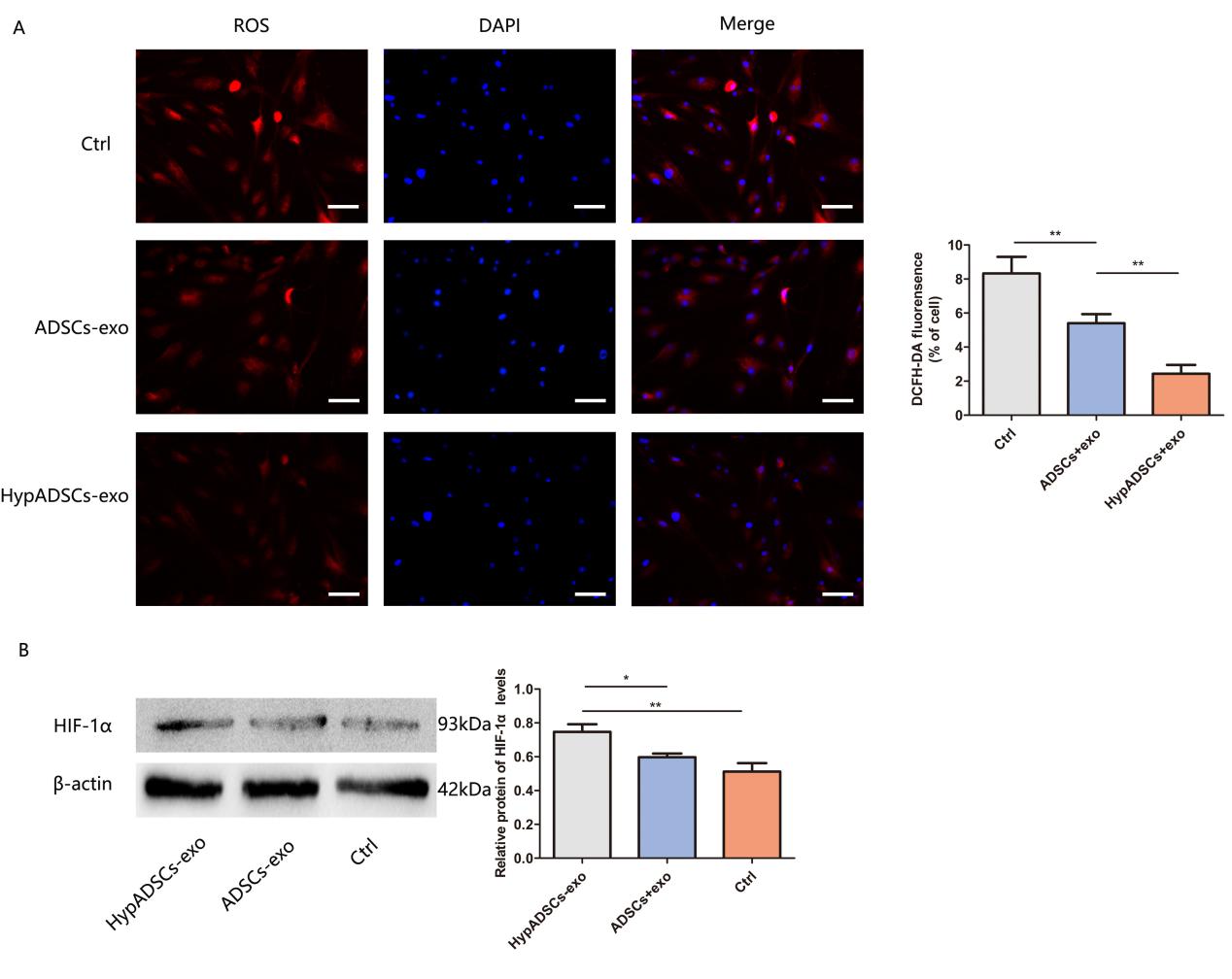


**Additional file 1: Figure S1.** (A) ROS generation was evaluated by fluorescence microscopy, and a stronger red fluorescence intensity indicates a higher production of ROS. (B) HIF-1α protein levels increased after HypADSCs-exo treatment of HF. Data are represented as mean ± SD. n = 3. *P < 0.05, **P < 0.01. Scale bars 100 μm.
