# Supplementary material for: Hypoxia adipose stem cell-derived exosomes promote high-quality healing of diabetic wound involves activation of PI3K/Akt pathways
Source: J Nanobiotechnology. 2021 Jul 7;19:202. doi: 10.1186/s12951-021-00942-0 (PMC8261989; doi:10.1186/s12951-021-00942-0)
Supplement: Supplementary file 2 — Additional file 2: Table S1. Primer sequences of the study. [file 12951_2021_942_MOESM2_ESM.docx]

**Table. 1 Primer sequences of the study**

**hADSCs Genes Forward primer Reverse primer**

**5ʹ→3’ 5ʹ→3’**

Actin-Mouse CACTGTCGAGTCGCGTCC TCATCCATGGCGAACTGGTG

Actin-Human TGGCACCCAGCACAATGAA CTAAGTCATAGTCCGCCTAGAA

bFGF-Human AAGAGCGACCCTCACATCAA ACGGTTAGCACACACTCCTT

COLⅠ-Human GAGGGCAACAGCAGGTTCACTTA TCAGCACCACCGATGTCCA

TGF-β-Human GTGAGCTAGATCGGTTGCTT CTTGCTAGATGGGAACTGAGAC

EGF-Human TCCAAGTGCATCAACACCGA GGGTGGAGTAGAGTCAAGACA

TGF-β-Mouse CGAAGCGGACTACTATGCTAAA TCCCGAATGTCTGACGTATTG

VEGF-Mouse AGGCTGCTGTAACGATGAAG TCTCCTATGTGCTGGCTTTG

COLⅠ-Mouse GCACGCCCAGTTTGGTAT TCACACAAGTCCCTATCCATTA

PDGF-Mouse TAACACCAGCAGCGTCAAGT TTCCCTACGCCTTCCTGTCT
